# Gastrointestinal Peptides as Therapeutic Targets to Mitigate Obesity and Metabolic Syndrome

**DOI:** 10.1007/s11892-020-01309-9

**Published:** 2020-05-21

**Authors:** Kleopatra Alexiadou, Tricia M-M. Tan

**Affiliations:** Department of Digestion, Metabolism and Reproduction, Imperial College London, London, UK

**Keywords:** Obesity, Type 2 diabetes, Gut hormones, GLP-1, Oxyntomodulin, PYY, GIP

## Abstract

**Purpose of Review:**

Obesity affects over than 600 million adults worldwide resulting in multi-organ complications and major socioeconomic impact. The purpose of this review is to summarise the physiological effects as well as the therapeutic implications of the gut hormones glucagon-like peptide-1 (GLP-1), oxyntomodulin, peptide YY (PYY), and glucose-dependent insulinotropic peptide (GIP) in the treatment of obesity and type 2 diabetes.

**Recent Findings:**

Clinical trials have proven that the widely used GLP-1 analogues have pleotropic effects beyond those on weight and glucose metabolism and appear to confer favourable cardiovascular and renal outcomes. However, GLP-1 analogues alone do not deliver sufficient efficacy for the treatment of obesity, being limited by their dose-dependent gastrointestinal side effects. Novel dual agonists for GLP-1/glucagon and GLP-1/GIP are being developed by the pharmaceutical industry and have demonstrated some promising results for weight loss and improvement in glycaemia over and above GLP-1 analogues. Triagonists (for example GLP-1/GIP/glucagon) are currently in pre-clinical or early clinical development.

**Summary:**

Gastrointestinal hormones possess complementary effects on appetite, energy expenditure, and glucose metabolism. We highlight the idea that combinations of these hormones may represent the way forward in obesity and diabetes therapeutics.

## Introduction

Obesity affects approximately 39% of the world’s adult population which represents a threefold increase since 1975 [1]; more than 1.9 billion adults were overweight in 2016 with emerging data from the USA estimating that by 2030 one in two adults will be obese [2].

Obesity is a multi-systemic disease state with several associated complications such as musculoskeletal issues, obstructive sleep apnoea, cardiovascular disease, infertility, and cancer (endometrial, breast, ovarian, prostate, liver, gallbladder, kidney, and colon) as well as significant metabolic sequelae, the most important being type 2 diabetes due to its high morbidity and mortality through micro- and macro-vascular complications such as ischaemic heart disease, stroke, and peripheral vascular disease [3, 4].

The most effective treatment for obesity and diabetes remission is bariatric surgery with lifestyle intervention and pharmacotherapy being moderately efficacious [5, 6]. However, bariatric surgery is not available to everyone due to limited availability of specialist surgeons and facilities, patient fitness for surgery, and patient choice [7]. Therefore, there is an unmet need for new and effective treatments for obesity and type 2 diabetes.

Lessons from bariatric surgery have clearly demonstrated that gut hormones are the key players mediating surgery’s favourable effects on weight loss and glucose homeostasis, hence making them a promising target for pharmacotherapy [8, 9]. The current review aims to illustrate the effects of the native peptides on weight and glucose homeostasis and present some data on the currently available and under development gut hormone analogues and will conclude with some future perspectives.

## Glucagon-Like Peptide-1 (GLP-1): Trailblazer for Gut Hormone Therapies

**Fig. 1:**
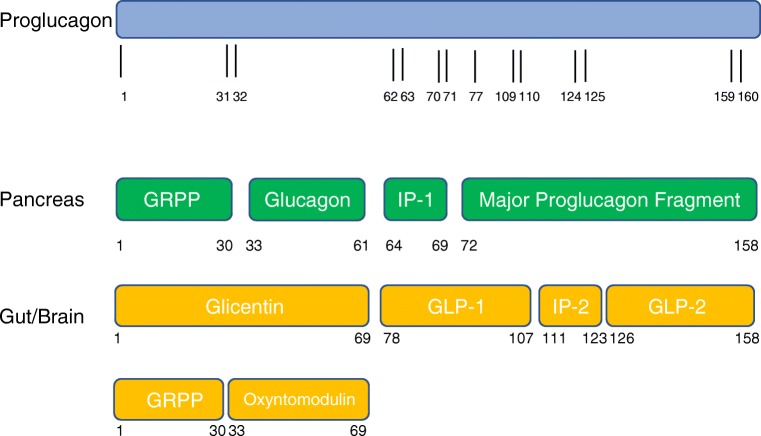
Differential post-translational processing of the proglucagon peptide. GRPP: glicentin-related pancreatic polypeptide; IP-1: intervening peptide-1; GLP-1: glucagon-like peptide-1; IP-2: intervening peptide-2; GLP-2: glucagon-like peptide-2

Glucagon-like peptide 1 (GLP-1) is a 30 amino acid peptide secreted from the enteroendocrine l-cells in response to ingestion of nutrients. It is a product of the post-translational processing of the proglucagon peptide in the l-cells by proconvertase 1 as opposed to the alternative post-translational processing by proconvertase 2 which releases glucagon in pancreatic α cells (Fig. 1). After release, it is rapidly degraded in plasma by the enzyme dipeptidyl-peptidase IV (DPP-IV). GLP-1 is the most extensively studied gut hormone with translational and clinical evidence for its efficacy [10]. Initial studies focused on the role of GLP-1 as an incretin hormone, stimulating glucose-dependent insulin secretion from β cells upon nutrient ingestion [11]. Subsequent studies showed the favourable effect of GLP-1 on pancreatic islets as a stimulant for β cell proliferation and survival as well as its inhibitory effect on α cells and glucagon secretion, likely through paracrine mechanisms such as inhibitory factors released from β cells and through stimulation of local δ cell somatostatin secretion [12]. Activation of the GLP-1 receptor leads to inhibition of gastric emptying and small bowel motility leading to a slower absorption of nutrients. This action is subject to tachyphylaxis which is detectable within hours [13]. Acute and sustained GLP-1 receptor signalling leads to reduction in appetite and food intake leading to subsequent weight loss [14, 15]. Emerging data suggest that GLP-1 has pleiotropic effects beyond appetite suppression and weight loss with additional cardioprotective and anti-inflammatory roles [16].

GLP-1 analogues have been developed over the last 20 years that incorporate DPP-IV resistance and other modifications that enable convenient daily or even weekly dosing. The first approved GLP-1 analogue for clinical use was exenatide in 2005, a peptide originally isolated from *Heloderma suspectum* lizard venom by John Eng in 1992 [17]. Since then, other GLP-1 analogues have been approved including lixisenatide, liraglutide, dulaglutide, albiglutide, and semaglutide. Long-lasting preparations such as exenatide LAR, dulaglutide, and semaglutide enable the effective treatment of type 2 diabetes with one injection a week. In a series of phase 3 trials (LEAD trials), liraglutide was shown to be more effective in reducing HbA1c by 1.2 to 1.6% when used either as monotherapy or in combination with other diabetes drugs, whilst conferring weight loss of 2–3 kg, an effect which is especially favourable in comparison to the weight gain associated with insulin glargine or sulphonylurea treatment [18]. The SUSTAIN-1 phase 3 trial demonstrated the safety and tolerability of once-weekly semaglutide injection as monotherapy in patients with type 2 diabetes, achieving reduction in HbA1c of 1.55% at the highest dose of semaglutide, 1 mg vs. 0.02% with placebo. Semaglutide 1 mg weekly was associated with a placebo-subtracted weight loss of 3.5 kg [19] after 30 weeks. Similar improvements in glycaemia and weight have been obtained with the other GLP-1 analogues and these are now firmly established in treatment pathways for diabetes, especially when priority is being given to weight loss [20]. The next foreseeable development on this frontier will be the introduction of oral semaglutide [21] which promises to extend the benefits of GLP-1 therapy to a greater population of people with diabetes.

Given that GLP-1 analogues were associated with appreciable weight loss effects in people with diabetes, it was natural to examine their effects in people with obesity. The SCALE trial examined the effect of higher doses of liraglutide (3 mg once daily vs. placebo) in obesity as a primary indication. At week 56, patients in the liraglutide group had lost a mean of 8.4 ± 7.3 kg of body weight, whereas those in the placebo group had lost a mean of 2.8 ± 6.5 kg (mean difference of − 5.6 kg) [22]. In a phase 2 dose-ranging randomised controlled trial, semaglutide, at doses up to 0.4 mg per day, achieved significantly greater weight loss compared with liraglutide and placebo: 13.8% at the 0.4 mg/day dose vs. 11.2% with liraglutide 3 mg per day and 2.3% for placebo. However, this enhanced efficacy was at the expense of adverse events, mainly nausea, diarrhoea, constipation, and vomiting; 15% of subjects given the highest dose discontinued the drug due to at least one adverse event as compared with 9% for liraglutide and 3% of placebo-treated subjects [23].

GLP-1 has several salutary effects on the cardiovascular system, including anti-inflammatory effects, cardioprotection during ischaemia, natriuresis and diuresis, decreased platelet aggregation, and reduction in post-prandial lipid production [24]. The LEADER trial specifically examined the effect of liraglutide at up to 1.8 mg in people with diabetes at high cardiovascular risk. The liraglutide group had favourable cardiovascular outcomes with a lower risk for the primary composite outcome of the first occurrence of death from cardiovascular causes, nonfatal myocardial infarction, or nonfatal stroke in comparison with placebo [25]. The SUSTAIN-6 trial examined the effect of once-weekly semaglutide (0.5 or 1 mg) on cardiovascular outcomes in people with type 2 diabetes and again showed a favourable effect on cardiovascular events compared with placebo [26]. Multiple cardiovascular outcome trials (ELIXA—lixisenatide, LEADER—liraglutide, SUSTAIN-6—semaglutide, EXSCEL—exenatide, Harmony Outcomes—albiglutide, REWIND—dulaglutide, and PIONEER-6—oral semaglutide) have reported, to a greater or lesser extent, improvements in cardiovascular outcomes, with a systematic review and meta-analysis of these trials involving a combined total of 56,004 participants showing that these drugs as a class reduced major adverse cardiovascular events by 12%, death from cardiovascular causes by 12%, fatal or nonfatal stroke by 16%, and fatal or nonfatal myocardial infarction by 9%. In addition, GLP-1 analogue treatment reduced all-cause mortality by 12%, hospital admission for heart failure by 9%, and a broad composite kidney outcome (development of new-onset macroalbuminuria, decline in estimated glomerular filtration rate [or increase in creatinine], progression to end-stage kidney disease, or death attributable to kidney causes) by 17%, mainly due to a reduction in urinary albumin excretion [27]. As a result, GLP-1 analogues are now recommended for patients with type 2 diabetes who are at high risk of cardiovascular disease [20].

In summary, GLP-1 analogues are mature clinical treatments for diabetes and obesity, with proven efficacy in reducing glycaemia, modestly reducing weight, and reducing cardiovascular events in people with type 2 diabetes at high risk of cardiovascular disease. Oral preparations are in the offing and will expand the appeal of these drugs to prescribers and patients. However, at the highest doses, people treated with GLP-1 analogues experience gastrointestinal side effects, which limits the magnitude of weight loss that can be obtained. Moreover, GLP-1 does not affect energy expenditure in humans [28] which means that this treatment does not compensate for the reduction in energy expenditure that is observed with weight loss, a phenomenon that again limits the weight loss associated with GLP-1 analogue treatment.

## Oxyntomodulin and the Concept of Dual GLP-1/Glucagon Agonism

To take the field of obesity and diabetes therapeutics beyond the proven benefits of GLP-1 analogue therapy, investigators have considered the use of GLP-1 in combination with other gut hormones that may bring complementary benefits. Glucagon is one such candidate: it has long been known that this peptide has insulinotropic effects, reduces food intake, and increases energy expenditure. Moreover, glucagon increases fat oxidation and there is interest in its effects in ameliorating fatty liver disease [29]. However, glucagon has the undesired effect of provoking hyperglycaemia [30].

Proof-of-concept physiological studies have shown that co-administration of GLP-1 and glucagon reduces food intake synergistically and increases energy expenditure. Importantly, GLP-1 ameliorates the hyperglycaemia which is provoked by glucagon [31, 32]. A natural dual GLP-1/glucagon agonist exists in the shape of oxyntomodulin, a 37 amino acid peptide which is co-secreted with GLP-1 from the l-cells of the small intestine as an additional product of the differential processing of proglucagon in the gut (see Fig. 1) [33]. Oxyntomodulin reduces food intake and increases energy expenditure, leading to significant weight loss in a 28-day clinical study in human volunteers over 28 days [34], and has been shown to improve insulin secretion in short-term clinical studies in people with diabetes [35]. However, the short half-life and rapid renal clearance of native oxyntomodulin hinder its development as a long-term treatment for obesity.

Based on the foregoing data, GLP-1/glucagon receptor agonists are under development. Cotadutide is a balanced GLP-1/glucagon dual agonist which has been shown in phase 2 trials in people with diabetes and obesity to significantly improve and to reduce body weight by 2–3 kg in comparison to placebo [36, 37]. Another GLP-1/glucagon dual agonist, SAR425899, has been shown in phase 1 trials to reduce HbA1c by up to 0.75% and body weight by up to 5 kg or so [38]. Further trials are required in order to establish whether these dual GLP-1/glucagon agonists are superior to GLP-1 analogues, and whether the glucagon activity might reverse some of the improvements in glycaemia seen with GLP-1 analogues alone.

## Peptide YY as an Appetite Suppressant

Peptide YY (PYY) is a 36 amino acid gastrointestinal hormone which is co-secreted from the l-cells together with GLP-1 and oxyntomodulin. The PYY(3-36) peptide is derived from the full-length PYY(1-36) via processing by the enzyme DPP-IV and binds to the neuropeptide Y (NPY) Y2 and Y5 receptors. PYY(3-36) exhibits an anorexic affect through Y2 receptors in the arcuate nucleus and infusion of PYY(3-36) in human volunteers induces a 33% reduction in food intake over 24 h [39]. In contrast to GLP-1 and oxyntomodulin, however, PYY(3-36) does not seem to have a marked effect on glucose-stimulated insulin secretion when given to human volunteers [40].

Studies using the combination of PYY(3-36) and GLP-1 showed an additive effect in the reduction of food intake in humans [41]. Functional MRI imaging studies show that brain areas implicated in appetite and interest in food are activated when subjects are shown pictures of food. PYY(3-36) and GLP-1 given individually reduce the activation of these areas. The co-infusion of PYY(3-36) and GLP-1 led to a synergistic effect with near-suppression of these areas, correlating with the suppression in appetite as assessed by food intake studies [42]. Co-administration of PYY(3-36) and oxyntomodulin had also an additive effect on the reduction in food intake in humans compared with each peptide alone [43].

PYY(3-36) itself has been studied as a monotherapy for weight loss when delivered nasally in human volunteers, but the efficacy in terms of weight loss was limited by dose-dependent adverse events in the form of nausea and vomiting [44], possibly because this preparation and method of delivery led to a rapid release of PYY(3-36) to supraphysiological levels. This contrasts with our experience in infusion studies where the PYY(3-36) levels are increased more slowly and to less marked peaks, leading to better tolerability [39]. PYY analogues continue to be developed to address the narrow therapeutic range of PYY-based drugs with slower release profiles to evade the adverse effects of nausea and vomiting [45] and as weekly injectable treatments such as Novo Nordisk’s PYY1875 [46]. Considering these factors, PYY analogues will likely be employed in future in combination with GLP-1 or other gut hormones, to enhance weight loss.

## Glucose-Dependent Insulinotropic Peptide (GIP): The ‘Twincretin’ Combination with GLP-1

Glucose-dependent insulinotropic polypeptide (GIP) is a 42 amino acid peptide which is synthesised and secreted from neuroendocrine K cells in the duodenum and jejunum in response to nutrient ingestion. Along with GLP-1, GIP is responsible for the incretin effect, namely the glucose-dependent stimulation of insulin secretion as a response to nutrient ingestion. GIP appears to have a bifunctional ‘stabiliser’ effect on glucose levels: under hyperglycaemic conditions, it potentiates insulin release whilst not affecting glucagon release, whereas under hypoglycaemic conditions, it increases glucagon release and does not affect insulin secretion [47]. However, GIP possesses other properties which make it unpromising as a therapy for diabetes and obesity. Firstly, under conditions of insulin resistance, such as obesity and type 2 diabetes, GIP promotes lipid deposition in subcutaneous adipocytes [48]. Secondly, GIP does not reduce appetite [49]. Thirdly, GIP’s actions on glycaemia seem to be impaired in conditions of chronic hyperglycaemia, which is the principal reason why GIP analogues have not developed for T2D. Indeed, some groups have explored the idea of GIP antagonism to reverse the stimulatory effect of GIP on glucagon secretion and increased fat deposition. The naturally occurring peptide GIP(3-30)NH_2_ has been successfully used as an GIP antagonist in human studies, inhibiting the incretin effect of GIP as well as its effect on glucagon secretion [50, 51], but the therapeutic potential of GIP antagonism is still not clear.

Interestingly, normalisation of glucose levels using insulin restores the incretin properties of GIP in diabetes [52]. The above data suggest that there is scope for a GLP-1 and GIP dual agonist combination (the so-called twincretin concept), where the former would restore euglycemia, permitting the latter to exert its full insulinotropic potential, thus synergistically improving glucose levels. Short-term infusion studies utilising the GLP-1/GIP combination compared with single GLP-1 infusion have failed to show an advantage of the combination over GLP-1 alone [49, 53, 54]. Nevertheless, pharmaceutical companies have pursued the ‘twincretin’ concept, developing GLP-1/GIP dual agonists such as tirzepatide, an analogue biased in favour of GIP over GLP-1 activity [55], and NNC0090-2746, another analogue with balanced GLP-1 and GIP activity [56]. Intriguingly, a phase 2B clinical trial of tirzepatide in people with diabetes and obesity suggested that this analogue, at the highest doses tested, has superior effects on glycaemia in comparison to the benchmark GLP-1 analogue dulaglutide (reductions in HbA1c of 1.9% vs. 1.2% when given for 26 weeks) as well as weight loss (reductions of 11.3 kg vs. 2.7 kg) albeit with a worse gastrointestinal side effect profile [57]. The phase 2a trials of NNC0090-2746 in people with diabetes and obesity also showed significant improvements in HbA1c over placebo with a placebo-subtracted reduction of 0.96% over 12 weeks’ treatment, as well as placebo-subtracted reductions in weight of up to 1.8% [56]. GLP-1/GIP twincretins therefore show some promise at this stage.

## Triagonism: Is Three Better than Two?

It is theoretically possible that, by blending three complementary gut hormone actions, we may be able to obtain enhanced efficacies over dual agonism. The physiological changes from bariatric surgery may serve as a model for this concept. Surgery, and in particular Roux-en-Y gastric bypass (RYGB), exerts many of its beneficial effects by activating the exaggerated release of GLP-1, oxyntomodulin, and peptide YY after eating, leading to improvements in glucose metabolism, suppression of appetite, and reductions in body weight [8, 9]. Building on this observation, we used a subcutaneous infusion pump to deliver a combination of GLP-1/oxyntomodulin/PYY(3-36) (GOP) at doses to replicate the elevated post-prandial levels found after bariatric surgery [58]. When administered for up to 12 h per day for 28 days in obese people with pre-diabetes/type 2 diabetes, we showed that GOP infusion achieves superior glucose tolerance and reduced glucose variability compared with RYGB and has a favourable effect on body weight. Our proof-of-concept study suggests that triple agonism of the GLP-1, glucagon, and Y2 receptors using the GOP combination may possess advantages even over RYGB, hitherto considered the standard-of-care for treatment of obesity and diabetes, and further studies are planned to explore the doses and combinations to obtain optimal efficacies [59].

Other groups have explored the concept of GLP-1/GIP/glucagon triagonism to obtain extra benefits from adding the benefits of glucagon action (promotion of energy expenditure and amelioration of fatty liver disease) to the ‘twincretin’ effects of GLP-1 and GIP. These properties were combined in the design of the unimolecular triagonist MAR423 with promising results in animal models which have been taken forward to an ongoing phase 1 trial [60]. HM15211 is another triagonist compound which has shown favourable pre-clinical data in animal models of steatohepatitis [61–63] and which is currently in phase 1 trials.

## Conclusions

**Fig. 2:**
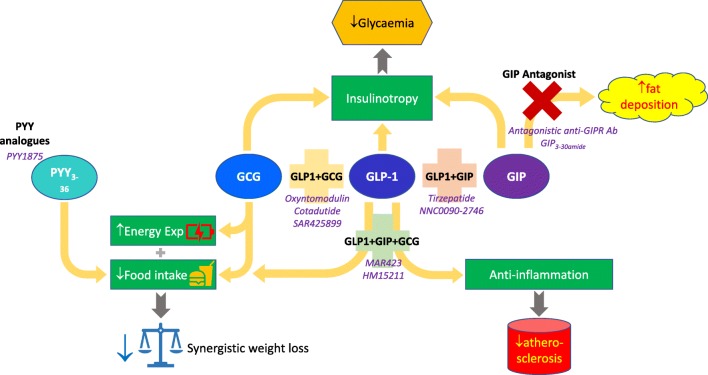
Complementary actions of gut hormone receptors combine to achieve desirable therapeutic outcomes. GCG: glucagon; GIP: glucose-dependent insulinotropic polypeptide; GLP-1: glucagon-like peptide-1; PYY: peptide YY

GLP-1 and its analogues have been the trailblazers for the concept of gut hormone therapy of diabetes and obesity. However, the dimensions of the global obesity and diabetes pandemics call for the development of new, effective, practical, and scalable treatments which will overcome the limitations of the GLP-1 analogues. Dual and triple hormone combinations are emerging as a promising strategy for such development (Fig. 2), but it seems fair to say that academic researchers and pharmaceutical industry have not yet struck on the optimal combination for treatment at this present moment. Moreover, even if the current dual and triple agonists successfully make it to market, it remains unknown whether they will inherit the same cardiovascular and renal benefits that have been identified for GLP-1. We await the data that will help us to identify such optimal combinations to achieve the promise of enhanced efficacy for weight loss and improvements in glycaemia without undue side effects.
